# Quantization and its breakdown in a Hubbard–Thouless pump

**DOI:** 10.1038/s41567-023-02145-w

**Published:** 2023-07-24

**Authors:** Anne-Sophie Walter, Zijie Zhu, Marius Gächter, Joaquín Minguzzi, Stephan Roschinski, Kilian Sandholzer, Konrad Viebahn, Tilman Esslinger

**Affiliations:** Institute for Quantum Electronics & Quantum Center, ETH Zurich, Zurich, Switzerland

**Keywords:** Ultracold gases, Topological matter

## Abstract

Geometric properties of wave functions can explain the appearance of topological invariants in many condensed-matter and quantum systems^1^. For example, topological invariants describe the plateaux observed in the quantized Hall effect and the pumped charge in its dynamic analogue—the Thouless pump^2–4^. However, the presence of interparticle interactions can affect the topology of a material, invalidating the idealized formulation in terms of Bloch waves. Despite pioneering experiments in different platforms^5–9^, the study of topological matter under variations in interparticle interactions has proven challenging^10^. Here we experimentally realize a topological Thouless pump with fully tuneable Hubbard interactions in an optical lattice and observe regimes with robust pumping, as well as an interaction-induced breakdown. We confirm the pump’s robustness against interactions that are smaller than the protecting gap for both repulsive and attractive interactions. Furthermore, we identify that bound pairs of fermions are responsible for quantized transport at strongly attractive interactions. However, for strong repulsive interactions, topological pumping breaks down, but we show how to reinstate it by modifying the pump trajectory. Our results will prove useful for further investigations of interacting topological matter^10^, including edge effects^11^ and interaction-induced topological phases^12–15^.

## Abstract (web summary)

Thouless pumping is the quantization of charge transport through the adiabatic variation of a system’s parameters. The robustness and breakdown of pumping under variations in interparticle interactions have now been shown with ultracold atoms in an optical lattice.

## Main

Ultracold quantum gases provide a versatile platform for investigating topological phenomena^16–18^, in which atoms take on the role of mobile charges. Although atoms are electrically neutral, effective magnetic fields can be generated via periodic modulation. However, the simultaneous presence of interactions and periodic driving often leads to detrimental energy absorption and population of highly excited modes^4,19,20^. Most experiments have so far been restricted to the non-interacting regime^21–24^ or the interactions remained fixed^8,25^. Conversely, realizing a many-body system with topology and variable interactions is still a challenge, despite substantial and ongoing theoretical interest^12–15,26–43^.

In our experiment, we create a dynamically tuneable superlattice by overlaying phase-controlled standing waves with an additional running-wave component and study topological charge pumping in the periodically driven, interacting Rice–Mele model^44^:

1 $$\begin{array}{lll}\hat{H}(\tau )&=&-\mathop{\sum}\limits_{j,\sigma }\left[t+{(-1)}{\,}^{j}\delta (\tau )\right]\left({\hat{c}}_{j\sigma }^{{\dagger} }{\hat{c}}_{j+1\sigma }+{{{\rm{h.c.}}}}\right)\\ &&+\,{{\varDelta }}(\tau )\mathop{\sum}\limits_{j,\sigma }{(-1)}{\,}^{j}{\hat{c}}_{j\sigma }^{{\dagger} }{\hat{c}}_{j\sigma }+U\mathop{\sum}\limits_{j}{\hat{n}}_{j\uparrow }{\hat{n}}_{j\downarrow }\end{array}.$$

**Extended Data Fig. 1:**
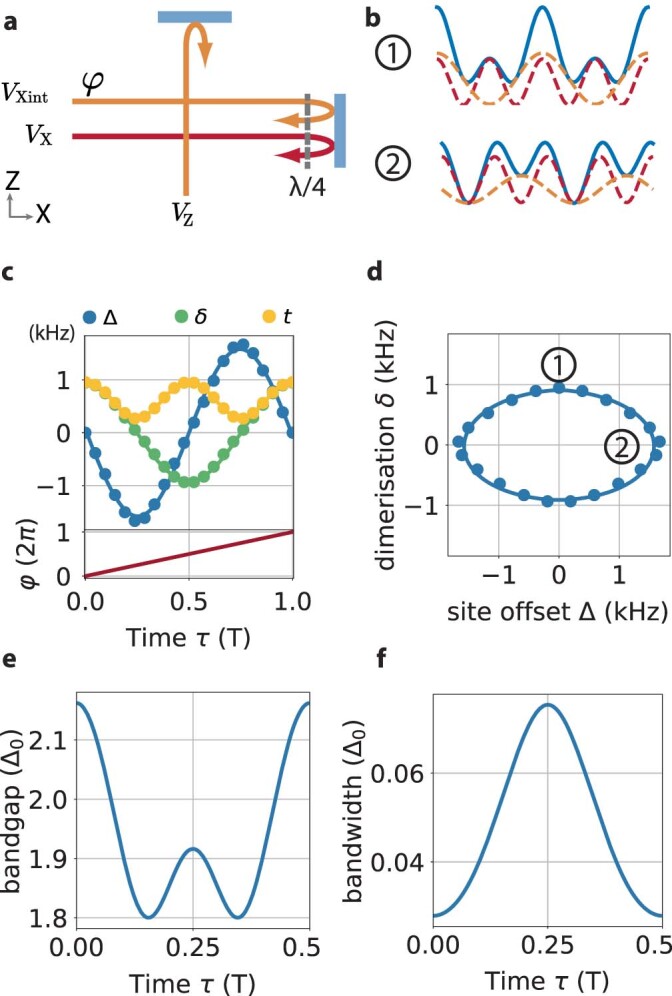
Optical lattice setup and Rice-Mele parameters. **a**, schematic of the optical setup in the *x*–*z* plane. The *λ*/4 waveplate produces an intensity imbalance between the different contributing beams in such a way that the phase *φ* can be used to move the chequerboard over the square lattice, realising pumping. **b**, idealised cut through the lattice potential in *x*-direction, corresponding to the two points along the pump cycle shown in **d**. The site-offset case (2) is exaggerated for clarity. **c**, tight-binding parameters during one pump cycle. The phase *φ* (defined in Eq. 2) is ramped from 0 to 2*π*. In the main text the time dependence of the average tunnelling *t* has been dropped for clarity. In contrast to previous realisations of the Rice-Mele pump with cold atoms^23,25^, the site offset Δ and dimerisation *δ* follow a sinusoidal waveform over a pump cycle (solid lines corresponds to the fitted sinusoid and the corresponding parameters are summarized in Table 1. The single-particle gap is dominated by the dimerised tunneling at *τ* = 0 and by the site offset at *τ* = *T*/4. **d**, elliptical trajectory of *δ* and Δ over one pump cycle. The solid line corresponds to the fitted curves in **c**. Single-particle band gap (**e**) and bandwidth (**f**) in units of Δ_0_ over a pump half-cycle (the second half is symmetric).

The interactions enter as the Hubbard *U* for two fermions of opposite spin *σ* ∈ {*↑*, *↓*} occupying the same lattice site *j*. The fermionic annihilation and number operators are denoted by ${\hat{c}}_{j\sigma }$ and ${\hat{n}}_{j\sigma }$, respectively. Both bond dimerization *δ*(*τ*) and sublattice site offset *Δ*(*τ*) are sinusoidally varied in time *τ* with period *T*, but out of phase with respect to each other. This cyclic and adiabatic modulation describes a quantum pump, which manifests itself in a drift of the many-body polarization^45^. For an insulator or a homogeneously filled band of free fermions (*U* = 0), this drift is quantized, realizing a Thouless pump^2^, protected by the single-particle gap of the bipartite lattice structure (equation (1), Extended Data Fig. 1 and Methods). During pumping, our system remains in the low-energy sector described by equation (1). For finite interactions (*U* ≠ 0), the Rice–Mele model encompasses a rich many-body phase diagram^46^, including the ionic Hubbard model with maximum site offset *Δ*_0_ and no dimerization^47,48^, as well as the interacting Su–Schrieffer–Heeger model with maximum dimerization *δ*_0_ and zero site offset^49^.

**Fig. 1:**
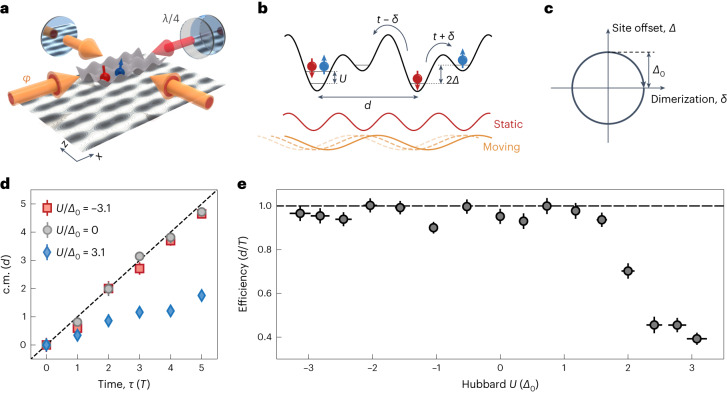
Topological pumping in the interacting Rice–Mele model. **a**, Schematic of the dynamical optical superlattice setup. The interfering lattice (yellow) is imbalanced along the *x* direction, leading to a movement of the ‘long’ lattice with respect to the ‘short’ lattice (non-interfering, red arrows) when ramping the phase *φ* of incoming light. The running-wave component is due to a rotated polarization of the retro-reflected laser beam (*λ*/4 waveplate). The standing wave in the *y* direction is not shown for clarity. Here *d* = *λ* = 1,064 nm is the size of one unit cell. **b**, Resulting lattice structure along *x* corresponds to the interacting Rice–Mele Hamiltonian (equation (1)). **c**, Sketch of the pumping trajectory in the parameter space spanned by site offset *Δ* and dimerization *δ*. Here *Δ*_0_ corresponds to half of the gap in the ionic Hubbard model (*δ* = 0). **d**, Measured in situ c.m. position of the fermionic cloud within five pumping cycles for *U*/*Δ*_0_ = {–3.1(2), 0, 3.1(2)} (red, grey and blue data points, respectively). Attractive and non-interacting atoms exhibit quantized pumping (black dashed line), whereas the movement of the repulsive cloud is strongly reduced. The data points and error bars correspond to the mean and standard error of eight individual measurements. **e**, Measured pumping efficiency (fitted slopes of **b**, averaged over the pumping direction) as a function of Hubbard *U*. Nearly quantized pumping efficiency persists for weakly interacting atoms (both attractive and repulsive) and strongly attractive interactions up to |*U*| = 3.1(2)*Δ*_0_ = 9.2(3)*t*. In the strongly repulsive regime, topological pumping breaks down. The error bars in the *y* direction correspond to the propagated error estimated from the uncertainty of the fit and those in the *x* direction, to the propagated error from lattice fluctuations. All the measurements in this figure were taken at a fixed period of *T* = 41.5(1.5)*ℏ*/*t*. Source data

**Extended Data Fig. 2:**
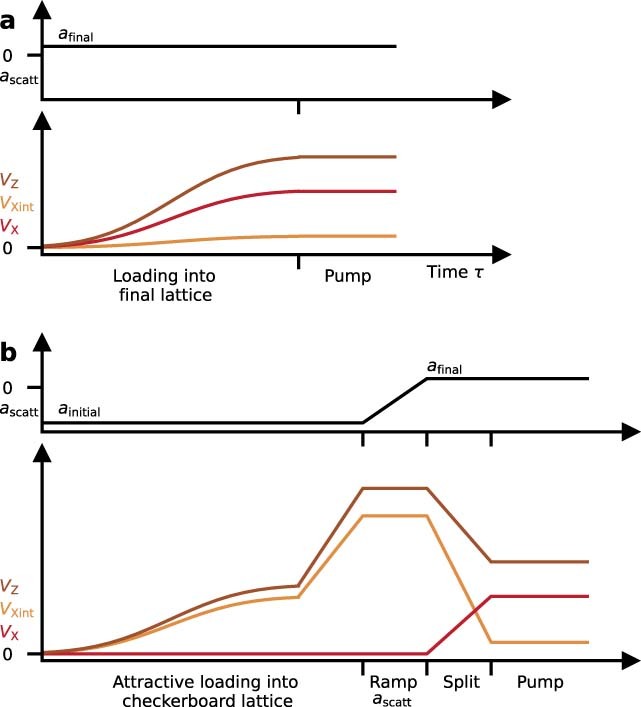
Lattice and interaction ramps for two different loading schemes. **a**, The ‘conventional’ lattice ramps with final depths [*V*_X_, *V*_Xint_, *V*_Y_, *V*_Z_] of [5.40(5), 0.09(2), 15.02(6), 17.04(8)]*E*_*R*_ are made up of s-shaped ramps at the final scattering length for the targeted *U*. **b**, The improved scheme includes an intermediate ramp into a deep checkerboard lattice at strongly attractive scattering lengths to maximise the fraction of atoms in half-filled unit cells. Intended interactions are then reached by ramping the magnetic field strength, for the − 9/2, − 7/2 mixture, or a ramp and a prior RF pulse for the − 9/2, − 5/2 mixture. Splitting the single cells of the checkerboard pattern in two then yields the final lattice depths [6.02(4), 0.37(3), 14.98(3), 17.0(3)]*E*_*R*_.

The experiments are performed using a balanced spin mixture (*↑*, *↓*) of ultracold potassium-40 atoms in a three-dimensional optical lattice (Fig. 1, Extended Data Fig. 1 and Methods). The total lattice potential comprises interfering laser beams in the *x*–*z* plane and additional non-interfering standing waves in all the three spatial directions, namely, *x*, *y* and *z* (ref. ^50^). These potentials combine to form one-dimensional superlattices along *x*. The phase between the interfering (‘long’) lattice with respect to the non-interfering (‘short’) lattice along *x* is dynamically controlled, inspired by the self-oscillating mechanism discussed in another work^51^. This traces an elliptical path of the Rice–Mele parameters *δ* and *Δ* around the origin. In contrast to previous realizations in optical lattices^23,25^, our setup uses a single laser source at *λ* = 1,064 nm for all the lattice beams, avoiding wavelength-dependent phase shifts in the optical path. Before pumping, we use a loading scheme with an intermediate lattice to increase the fraction of atoms in doubly occupied unit cells. This fraction is characterized with an independent measurement, achieving approximately 80% in the strongly attractive regime, and around 50% in both weakly interacting and strongly repulsive regimes (Extended Data Fig. 2 and Methods).

**Extended Data Fig. 3:**
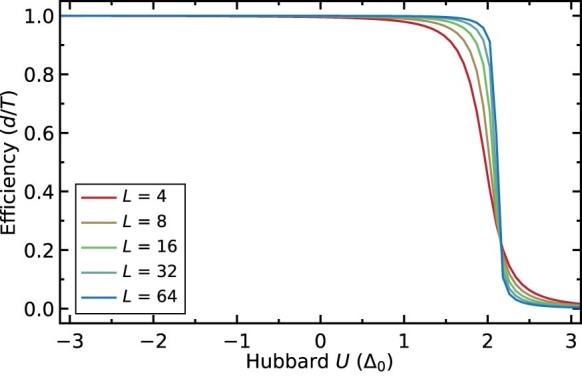
Effect of finite system size. Pumping efficiency vs. Hubbard *U* at different system size *L* in unit of lattice site, calculated with DMRG, assuming half-filling in OBC. The tight-binding parameters are chosen to be the same as in Fig. 1e. Smaller system sizes, which we expect from our loading scheme, only slightly change the position and steepness of the transition between the quantised and break down regime versus *U*. Source data

In a first experiment, we track the many-body polarization by measuring the centre-of-mass (c.m.) position of the atomic cloud within five pumping cycles for varying interaction strengths *U* (Fig. 1d). We choose the lattice such that the single-particle gap is approximately constant over such a pumping cycle and is given by 2*Δ*_0_. Fitting a line to these data yields the efficiency of the pump, which is plotted versus *U* (Fig. 1e). This measurement characterizes the topological behaviour of an interacting Thouless pump, allowing us to distinguish three cases. First, quantized pumping (Fig. 1e, black dashed line) persists for weak interactions (*U* ≲ *Δ*_0_ = 2.9*t*), both in the attractive and repulsive situations. We attribute deviations from unity to fluctuations in the position of our in situ atomic cloud and residual changes in momentum distribution owing to drifts in the filling fraction. Second, this plateau of nearly quantized topological transport, averaging to an efficiency of 0.96(3), extends to large attractive values of *U*, which exceed the single-particle gap 2*Δ*_0_. Increasingly large attractive Hubbard |*U*| leads to the formation of double occupancies (DOs)^52^. Topological pumping then relies on the transport of pairs, in contrast to the standard description of pumping with single atoms^39^. Importantly, we observe a clear asymmetry between strongly attractive and strongly repulsive interactions. Beyond *U* ≃ +2*Δ*_0_ = 5.8*t*, topological pumping breaks down and its efficiency decreases down to 0.39(3). For comparison, we perform numerical simulations of the many-body ground state at half-filling with a density matrix renormalization group (DMRG) algorithm on 4–64 lattice sites and find that the pumping efficiency drops to zero for large *U* (Extended Data Fig. 3). The remaining pumping efficiency of 0.4–0.5 for large repulsive interactions in the experimental data is a result of the non-zero fraction of atoms in singly occupied unit cells. This agrees with an independent measurement of the initial state, where 55(7)% of atoms are found in doubly occupied unit cells. Our observations, therefore, support the picture that topological pumping breaks down for doubly occupied unit cells at large repulsive *U*.

**Fig. 2:**
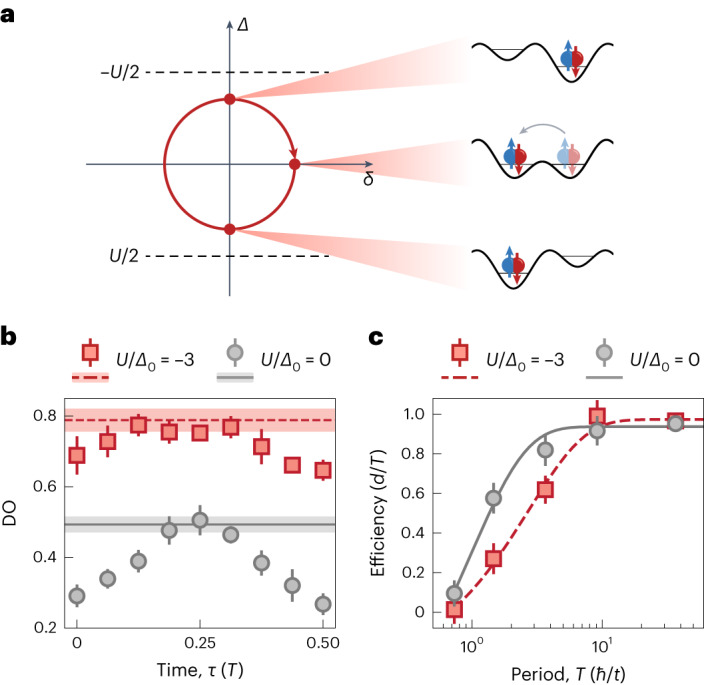
Quantized pumping of pairs in the attractive Rice–Mele model. **a**, In the strongly attractive regime, the pumping mechanism is a result of the tunnelling of pairs of fermions. **b**, Measured DO fraction over half a pumping cycle for *U*/*Δ*_0_ = 0 (grey points) and *U*/*Δ*_0_ = −3.0(1) (red squares). The large fraction of DOs and its small modulation over a pumping cycle for *U*/*Δ*_0_ = −3.0(1) compared with *U* = 0 supports the picture of pair pumping in the strongly attractive regime. The solid grey and dashed red lines indicate the maximum attainable DO fraction given by our lattice loading scheme. Each data point and error bar corresponds to the mean and standard error of six individual measurements split equally between the pumping directions. **c**, Adiabatic timescale of the topological pump for non-interacting and strongly attractive atoms. The measured efficiency is plotted versus pumping period in units of tunnelling times for *U* = −3.0(2)*Δ*_0_ = −9.2(3)*t* (red squares) and *U* = 0 (grey points). The data points correspond to the fitted slopes of the c.m. drift over two pumping cycles averaged over at least nine iterations and the pumping direction. The data point at *T* = 36.5*ℏ*/*t* is taken from the dataset for Fig. 1e at *U*/*Δ*_0_ = −3.1(2). The error bars correspond to the propagated error estimated from the uncertainty of the fit. Source data

**Extended Data Fig. 4:**
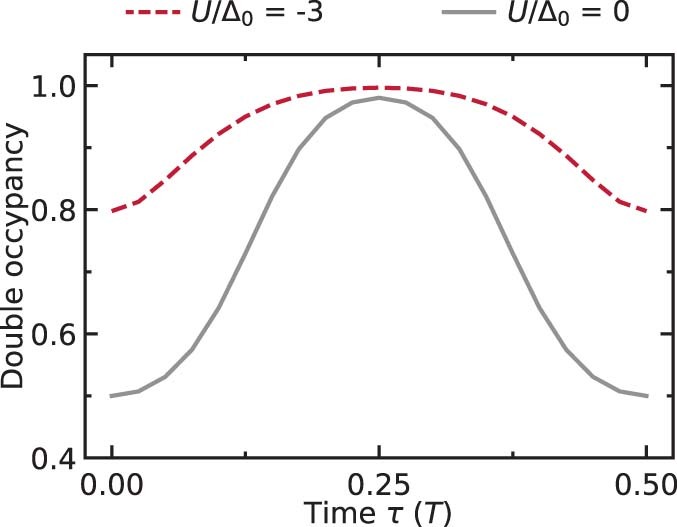
Numerical simulation of double occupancy fraction over half a pumping cycle with different Hubbard interactions *U*. The numerical simulations assume *L* = 64 lattice sites in OBC and half-filling. The tight-binding parameters are chosen to be the same as in Fig. 2b. Compared to the simulations, we record an overall lower double occupancy in the experiment (Fig. 2b) as a result of an overall average filling lower than one half. Source data

The observation of pair pumping for strong attractive interactions is substantiated by additional observables, including the evolution of DO fraction and the timescale for adiabaticity. We detect the fraction of pairs over half a trajectory for the non-interacting (*U* = 0, grey data points) and strongly attractive (*U*/*Δ*_0_ = −3.0(1), red data points) systems (Fig. 2b). The solid grey and dashed red lines (Fig. 2b) indicate the maximum accessible DO given by our lattice loading, which is equal to the fraction of the initially doubly occupied unit cells determined via an additional measurement (Methods). In the absence of interactions, the delocalization of atoms within a unit cell leads to a finite DO at *τ* = 0 and *Δ* = 0. A large negative *U* gives rise to an increased initial DO. Although the DO increases by more than 0.2 over the course of a cycle for *U* = 0 when reaching the maximum site offset *Δ*_0_, the high fraction in the attractive system only increases by half as much. Thus, we can conclude that the pairs for *U*/*Δ*_0_ = −3.0(1) largely remain bound over the pumping cycle. The residual modulation in DO over half the pumping cycle is also reflected by DMRG simulations (Extended Data Fig. 4). By analogy, quantized pumping should also be possible with repulsively bound pairs^12^ for *U* > 0, which we plan to investigate in the future.

The pumping of pairs also manifests itself in a change in adiabaticity timescale, compared with single atoms. Generally, the timescale for adiabatic following is determined by the minimum energy gap to the first excited state over a pumping cycle, which—in the non-interacting Rice–Mele model—corresponds to the second Bloch band of the bipartite lattice. In the experiment, the transport efficiency for the attractive pairs drops at longer periods compared with *U* = 0. In Fig. 2c, exponential fits to the data points yield 1/*e* times of 2.7(4)*ℏ*/*t* for *U*/*Δ*_0_ = −3.0(2) (dashed red line) and 1.0(2)*ℏ*/*t* for *U*/*Δ*_0_ = 0 (solid grey line). The increase in the adiabaticity timescale indicates that the energy gap becomes smaller in the attractive regime and agrees with the estimate 2*t*^2^/|*U*| ≃ 0.33(1)*t* (at *τ* = 0; Extended Data Fig. 1) for the effective tunnelling of hardcore bosons.

**Fig. 3:**
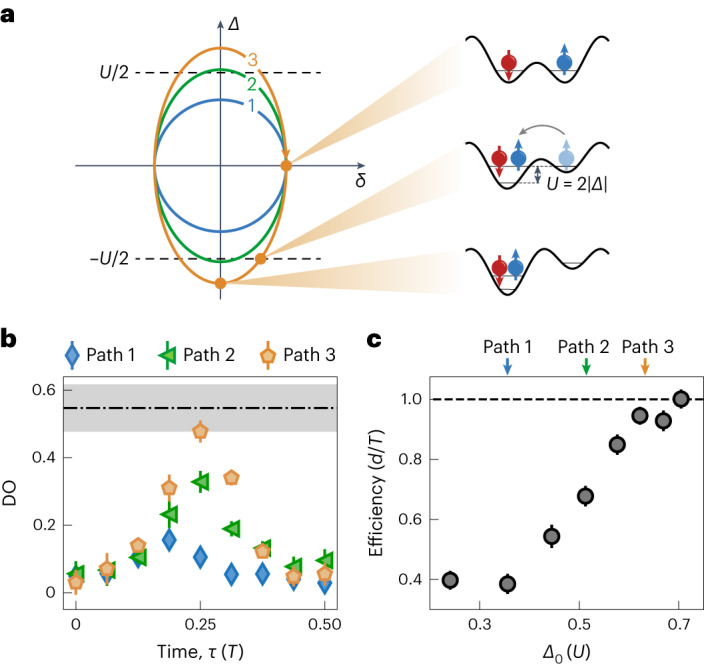
Quantized pumping via resonant tunnelling for strongly repulsive interactions (*U* ≃ 8*t*). **a**, Three pumping trajectories with varying maximum site offsets *Δ*_0_ and fixed maximum dimerization *δ*_0_. All the three paths start in the same state. The first trajectory (blue path) prohibits the formation of DOs and therefore precludes transport. Trajectories 2 and 3, with larger *Δ*_0_ (blue and green paths), allow for tunnelling between the sites of a unit cell when crossing the |*Δ*(*τ*)| = *U*/2 line parallel to the dimerization axis (black dashed lines). **b**, Measured DO fraction over half a pumping cycle for the three trajectories shown in **a**. **c**, Pumping efficiencies versus maximum site offset *Δ*_0_ for varying pumping paths. The efficiency increases from 0.40(3) to unity as *Δ*_0_ becomes large enough to allow for resonant tunnelling between a DO and localized single occupancies on each site. Data points and errors bars are obtained analogously to that in Fig. 2b,c. Source data

**Extended Data Fig. 5:**
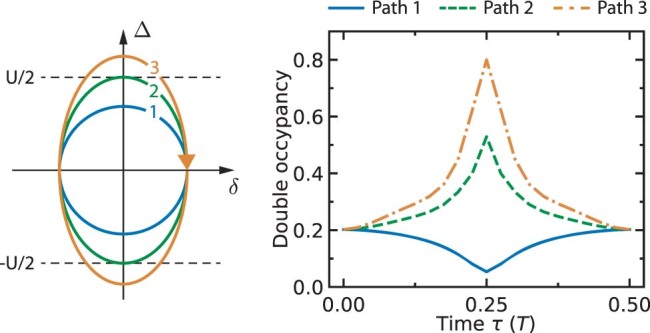
Numerical simulation of double occupancy (DO) fraction over half pumping cycle with different pumping trajectories. The numerical simulations assume *L* = 64 lattice sites in OBC and half-filling. The maximum site offset Δ_0_ for path 1,2,3 and the Hubbard *U* are chosen to be the same as in Fig. 3b. The experimental data (Fig. 3b) shows an overall lower double occupancy as a result of an average filling lower than one half. For path 1 the ground state simulation and experimental data do not exhibit the same behaviour versus *τ*. We attribute the difference to: (i) imperfect double occupancy detection around *τ* = 0 and *τ* = *T*/2. Due to a large tunnelling rate, the ramp to a square lattice for the DO detection (Methods) is not fast enough to completely freeze the dynamics of the atoms, which yields a slightly lower DO fraction than its actual value, (ii) at *τ* = *T*/4, the system can be characterized by an ionic Hubbard model, which exhibits gapless spin excitations. Therefore, the ideal adiabatic following of the instantaneous ground state is hindered, resulting in a lower than expected DO fraction after *τ* = *T*/4. Source data

Next, we investigate how to recover quantized transport in the strongly repulsive regime where pumping breaks down (Fig. 1d). To that end, we modify the pump trajectory and increase the maximum site offset *Δ*_0_ (Fig. 3a, paths 2 and 3), compared with the initial trajectory (path 1), whereas keeping the starting point and interactions fixed. Path 1 corresponds to the data point with the same absolute *U* in Fig. 1e (*U* = 2.8(1)*Δ*_0_ = 8.0(3)*t*). As a result of increasing *Δ*_0_, single occupancies and DOs become resonantly coupled by tunnelling^12^. Thus, an asymmetric charge distribution within a unit cell becomes energetically allowed. This asymmetry manifests in the change in polarization and is necessary for transport. We demonstrate this process in our experiment by measuring the DO fraction for all three trajectories (Fig. 3a) over half a pumping cycle (for paths 1 to 3, *Δ*_0_ = {0.35(1), 0.50(1), 0.61(1)}*U* with fixed *U*). The initial fraction is below 0.1 for all the paths considered here, reflecting the identical initialization. Although the DO for path 1 remains below 0.17, it reaches values of 0.33(3) and 0.48(2) for paths 2 and 3, respectively, as the line of *Δ* = *U*/2 is crossed and pair formation is restored. For path 3, the measured fraction even reaches the maximum possible value within error (Fig. 3b, black dash–dot line), determined by the initially doubly occupied unit cells (Methods). The observation is qualitatively consistent with numerical calculations (Extended Data Fig. 5). The influence of resonant pair formation on transport becomes clear with a measurement of efficiency versus maximum site offset *Δ*_0_ (Fig. 3c). For low values of *Δ*_0_, the pump efficiency is roughly constant at around 0.4. Increasing *Δ*_0_ leads to a growth in efficiency up to unity as the resonance condition for tunnelling is fulfilled.

In conclusion, we have experimentally characterized the topological properties of interacting Thouless pumps covering the full range of Hubbard *U*, from strongly attractive through intermediate to strongly repulsive. Remarkably, we observe a clear asymmetry between large attractive and large repulsive interactions. Although the robustness of quantized pumping of the former can be explained by an effective hardcore boson picture, the latter experiences a marked breakdown of transport. The experimental tools presented in this work also provide a pathway to study how interactions affect the role of spatial^10^ and temporal disorders, as well as edge physics^11^. Furthermore, our approach could enable topological transport that has no counterpart in the limit *U* → 0, leading to novel interaction-induced topological states^12–15^.

*Note added to proof: During the review of this manuscript, we became aware of related works*^53,54^.

## Methods

### Experimental sequence

We start by evaporatively precooling a cloud of fermionic ^40^K in the magnetic state *F* = 9/2, *m*_*F*_ = −9/2 and confining it to a crossed dipole trap. We then create a spin mixture of *m*_*F*_ = {−9/2, −7/2} and further evaporatively cool it, yielding 47,000(4,000) atoms at a temperature of 0.11(3)*T*/*T*_*F*_ (values in the brackets correspond to the standard deviation over all the measured data points; the atom number is calibrated within a systematic error of 10%). The atoms are subsequently loaded into a three-dimensional lattice within 200 ms. Using the magnetic Feshbach resonance at 202.1 G, we tune the *s*-wave scattering length between the atom in the −7/2 and −9/2 sublevels to be very strongly attractive, that is, *a* → −∞. Subsequently loading the atoms into a shallow chequerboard within 200 ms and then deep chequerboard within 10 ms leads to a high DO fraction^50^. To reach large repulsive interactions (*U*/*Δ*_0_ ≳ 1; Figs. 1d,e and 3), we then apply a radio-frequency (RF) sweep, transferring the atoms in the −7/2 to the −5/2 magnetic sublevel and keeping the −9/2 and −7/2 mixture otherwise. To reach the final lattice, we ramp the magnetic field to the scattering length, yielding the targeted *U* and split the sites of the chequerboard lattice into two sites along the *x* direction (Extended Data Fig. 2). This loading procedure results in many copies of the ground state of half-filled double wells (one of each spin in one unit cell). The improved loading procedure results in a larger fraction of atoms in doubly occupied unit cells and larger number of holes, which varies with the interaction strength during the split.

**Extended Data Fig. 6:**
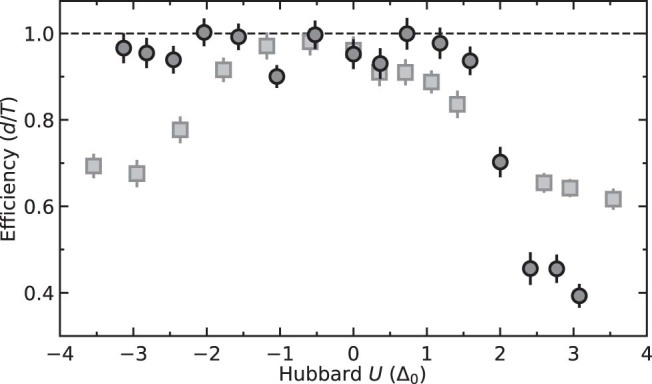
Pumping efficiency vs *U* for two different loading schemes and lattices. The pump efficiency with the improved loading scheme (dark circles, [Δ_0_, *δ*_0_, *t*] = [1750, 900, 625] Hz) exhibits a more pronounced plateau for attractive interactions, compared to conventional lattice loading (grey squares, [Δ_0_, *δ*_0_, *t*] = [847, 460, 403] Hz). The significant improvement of the signal on the attractive side is a result of both the new loading scheme, as well as a different final lattice configuration. In the improved lattice, the energy gap is roughly twice as large (3.6 kHz compared to 1.84 kHz in the conventional lattice) which reduces the higher band population during the loading. Likewise, the breakdown of transport on the repulsive side shows a steeper decline beyond a critical *U*, compared to conventional lattice loading. Error bars correspond to the propagated error estimated from the uncertainty of the linear fit. Source data

Compared with the previous loading scheme^56^, where the atoms were directly loaded into the lowest band of the final lattice, this fraction contributes to a larger breakdown signal for repulsive interactions (a comparison of pump efficiencies versus *U* is shown in Extended Data Fig. 6 and the DMRG calculations versus *U* in Extended Data Fig. 3 for different system sizes). Also, this preparation precludes the presence of atoms in the higher band and therefore a more persistent unity pumping efficiency on the attractive side. The resulting trapping frequencies are 86.1(1.1), 77.3(0.8) and 121.8(1.2) Hz in the *x*, *y* and *z* directions, respectively.

### Detection methods

After pumping the system for varying times, we either measure the in situ c.m. position of our cloud or detect the DO fraction.

#### Position of c.m

We detect the in situ c.m. position of our atomic cloud by taking an absorption image directly after the ramp of the phase, in the presence of the dipole trap, optical lattice and homogeneous magnetic field. The conversion from pixel size to lattice sites is done by independently measuring the displacement of the cloud in a lattice with a very large gap to the next excited band over 50 cycles in steps of 10.

#### DO fraction

For the DO fraction, we first freeze the dynamics of the atoms by quenching into a deep cubic lattice within 100 μs. We then sweep the magnetic field over the −7/2 and −9/2 Feshbach resonance and spectroscopically resolve the interaction shift with RF radiation, transferring atoms in the −7/2 (−5/2) state in doubly occupied sites to the −5/2 (−7/2) state. The Zeeman sublevels are then separated by applying a magnetic-field gradient and 8 ms time of flight^20^.

#### Fraction of atoms in half-filled unit cells

For the determination of fraction of atoms in doubly occupied unit cells, we take the sum of the DO, singlet and triplet fractions. As for the DO measurement, the detection of the latter two start with a freeze ramp into a deep cubic lattice. DOs are then eliminated by applying two (one) consecutive Landau–Zener sweeps, transferring the atoms in the −7/2 (−5/2) state to the −3/2 state: doubly occupied sites then host a very short lived −3/2 and −9/2 mixture, which is lost from the trap. A magnetic gradient leads to an oscillation between the two populations, and the extrema yield the singlet and triplet fractions. To measure these, the lattice is ramped to a chequerboard configuration, which merges adjacent sites. As a consequence of the Pauli exclusion principle, triplets are then converted to one atom in the lowest band and one in the higher, whereas singlets form DOs in the lowest band. These single occupancies or DOs are detected with the same method as previously described for DOs. For normalization of the fraction of atoms in half-filled unit cells, we take the number of atoms *N* from the same measurement as the one done to assess the number of DOs.

### Optical lattice

The lattice is made up of four retro-reflected beams at a wavelength of 1,064 nm. The non-interfering beams in the *x*, *y* and *z* directions create a cubic lattice to which the interfering beams in the *x*–*z* plane superimpose a chequerboard lattice. The resulting potential as seen by the atoms is given by

2 $$\begin{array}{ll}V(x,y,z)=&-{V}_{{{{\rm{X}}}}}{I}_{{{{\rm{self}}}}}{\cos }^{2}(kx+\vartheta /2)\\ &-{V}_{{{{\rm{Xint}}}}}{I}_{{{{\rm{self}}}}}{\cos }^{2}(kx)\\ &-{V}_{{{{\rm{Y}}}}}{\cos }^{2}(ky)\\ &-{V}_{{{{\rm{Z}}}}}{\cos }^{2}(kz)\\ &-\sqrt{{V}_{{{{\rm{Xint}}}}}{V}_{{{{\rm{Z}}}}}}\cos (kz)\cos (kx+\varphi )\\ &-{I}_{{{{\rm{XZ}}}}}\sqrt{{V}_{{{{\rm{Xint}}}}}{V}_{{{{\rm{Z}}}}}}\cos (kz)\cos (kx-\varphi )\,,\end{array}$$

where *k* = 2π/*λ*. The lattice depths [*V*_X_, *V*_Xint_, *V*_Y_, *V*_Z_] used in this paper are given by [6.02(4), 0.37(3), 14.98(3), 17.0(3)]*E*_R_, measured in units of recoil energy *E*_R_ = *h*^2^/2*mλ*^2^, where *m* is the mass of the atoms. The phase *φ*, which is the relative phase between the incoming lattice beams in the *x* and *z* directions, governs the depth and relative position of the chequerboard with respect to the square lattice. The angle *ϑ*, defining the relative position between the one-dimensional sinusoidal lattice formed by *V*_X_ and that formed by *V*_Xint_, is controlled by the difference in light frequency of the two beams. We calibrate *ϑ* to 1.000(2)π by minimizing the DO during splitting of a chequerboard into a dimerized lattice at *U* = 0. The imbalance factors *I*_self_ and *I*_XZ_ are due to the *λ*/4 waveplate in the retro-path (Fig. 1 and Extended Data Fig. 1). The factor *I*_XZ_ plays a crucial role in our pumping scheme, which is based on sliding a varying chequerboard lattice over a square lattice. The sliding is achieved by ramping the relative phase *φ*, which is stabilized using a locking scheme, as detailed in the next section. Without the imbalance (that is, *I*_XZ_ = 1), as was the case in our previous work^20^, the phase *φ* would enter as an overall amplitude cos(*φ*). However, in case of *I*_XZ_ < 1, the interference terms proportional to $\sqrt{{V}_{{{{\rm{Xint}}}}}{V}_{{{{\rm{Z}}}}}}$ in equation (2) acquire a *φ*-dependent position, explaining the ability to slide the chequerboard using *φ*. We rotated the *λ*/4 waveplate such that the incoming, linearly polarized light is rotated by 26° after passing the plate twice. This results in imbalance factors of *I*_self_ = 0.98(2) and *I*_XZ_ = 0.81(2), which are independently calibrated using lattice modulation spectroscopy.

The Rice–Mele parameters in equation (1) are calculated via the basis of maximally localized Wannier states, spanning the space of solutions to the single-particle Hamiltonian with potential equation (2). Overlap integrals between these Wannier states yield the relevant tight-binding tunnelling elements, on-site energies and interactions *U*. The values of *Δ*, *δ* and *t* are plotted in Extended Data Fig. 1c as a function of *φ* ∈ [0, 2π]. Typical parameters are *Δ*_0_ ≃ 3.0*t* and *δ*_0_ ≃ 1.5*t*, leading to small variations in the single-particle bandgap between 1.8*Δ*_0_ and 2.0*Δ*_0_ over one period. Sinusoidal fits to this data simplify the theoretical description; the resulting fit parameters are listed in Table 1. Due to the strong confinement along *y* and *z*, the tunnellings along those directions *t*_Y,Z_ are below 20 Hz over the whole pump cycle. The on-site interaction *U* is 995 Hz for a reference scattering length of 100 Bohr radii, which varies by about 3% over the pump cycle, and the interaction between neighbouring sites is always below 50 Hz.

**Table 1:** Rice–Mele parameters for the fitted sines

Parameter	Offset	Amplitude	Frequency	Phase offset
	*B* (Hz)	*A* (Hz)	*ν*	*κ*
*t*	625	340	2	π/2
*Δ*	0	1,750	1	π
*δ*	0	900	1	π/2

Extended Data Fig. 1 provides details of the fitted sines. The parameters correspond to the expression *B* + *A*sin(2π*ντ*/*T* + *κ*), where *τ* is the time and *T* is the pump period.

### Phase lock

**Extended Data Fig. 7:**
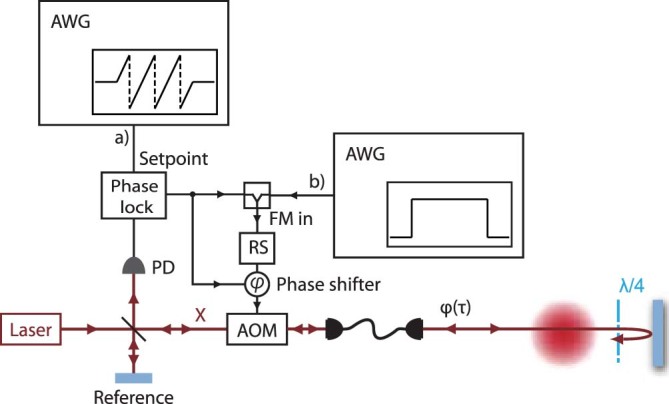
Schematic of the phase control. The back-reflected lattice beam forms a Michelson interferometer together with the reference path before the optical fibre. A linear increase in the interference phase can be realized either by linearly ramping the set-point (a) or by using a square waveform as input for the frequency modulation, which will also result in a linear phase ramp (b).

Topological pumping is realized by shifting the interference phase *φ* in time. Extended Data Fig. 7 illustrates the scheme for controlling *φ*, taking the *x* direction as an example. The setup is replicated on the *z* axis, which is not shown in Extended Data Fig. 7 for clarity. Active stabilization of the light phase is necessary since the optical fibre introduces considerable phase noise. In short, back-reflection from the optical lattice forms a Michelson interferometer together with a reference beam, which does not pass through an optical fibre. In this manner, the absolute phase of the lattice can be measured, assuming a perfectly stable reference arm. We shift the phase of the lattice beam by using the frequency modulation input of a Rohde & Schwarz function generator (SMC100A), creating the RF frequency for the acousto-optic modulator. A small frequency shift will result in a phase shift of the laser beam at the position of the atoms (Extended Data Fig. 7, red cloud). We additionally correct for small deviations to the absolute phase by shifting the phase of the output of the Rohde & Schwarz generator to the acousto-optic modulator with a phase shifter. The setpoint of the phase can now be varied in two different ways: for long pumping periods (longer than 5 ms), an arbitrary waveform generator (Keysight 33500B) generates a sawtooth signal as the setpoint of the phase lock, which results in a linear phase ramp. For short pumping cycles (less than 10 ms), the bandwidth of the phase lock is not large enough to follow the setpoint. In this case, the arbitrary waveform generator creates a square signal, which is added to the feedback signal from the phase lock before the frequency modulation input using a power splitter. The square waveform after integration also results in a linear phase shift of the lattice beam. For example, a frequency shift of 400 Hz on the RF signal of the acousto-optic modulator leads to a pumping slope of Δ*φ*/Δ*τ* = 2π/5 ms.

### DMRG calculations

Numerical results of pumping efficiency and DO dynamics presented are calculated with DMRG using the TeNPy Python package^55^ (version 0.6.1). The polarization and DO dynamics (Extended Data Figs. 3–5) are calculated using open-boundary conditions, where we assume *L* = 64 and half-filling (one of each spin in one unit cell). Throughout the calculation, we have selected the maximum bond dimension of *χ* = 100. The tight-binding parameters used in the simulation are identical to those used in the corresponding experiments. The polarization, that is, the c.m. of the ground state |*Ψ*(*t*)〉 is defined by

$${P}_{{{{\rm{open}}}}}(t)=\frac{1}{L}\mathop{\sum}\limits_{\sigma }\mathop{\sum }\limits_{j=0}^{L-1}\left\langle {{\varPsi }}(t)\right\vert (\,j-{j}_{0}){\hat{n}}_{j\sigma }\left\vert {{\varPsi }}(t)\right\rangle ,$$

and the DO fraction ${{{\mathcal{D}}}}$ is defined as the fraction of atoms on doubly occupied lattice sites as

$${{{\mathcal{D}}}}=\frac{2}{N}\mathop{\sum}\limits_{j}\langle {\hat{n}}_{j\uparrow }{\hat{n}}_{j\downarrow }\rangle ,$$

where *N* is the total atom number.

## Online content

Any methods, additional references, Nature Portfolio reporting summaries, source data, extended data, supplementary information, acknowledgements, peer review information; details of author contributions and competing interests; and statements of data and code availability are available at 10.1038/s41567-023-02145-w.

### Source data

Source Data Fig. 1 Experimental data with uncertainties.

Source Data Fig. 2 Experimental data with uncertainties.

Source Data Fig. 3 Experimental data with uncertainties.

Source Data Extended Data Fig. 3 Numerical simulation data.

Source Data Extended Data Fig. 4 Numerical simulation data.

Source Data Extended Data Fig. 5 Numerical simulation data.

Source Data Extended Data Fig. 6 Experimental data with uncertainties for the conventional loading scheme only. Data for the improved loading scheme are the same as Fig. 1e.

## Data Availability

Source data are provided with this paper. All data files are available from the corresponding authors on request.

## Code availability

Source codes for the data processing and numerical simulations are available from the corresponding authors upon request.
